# O.5.2-9 Development of an active lifestyle intervention to promote physical activity sustainably in stroke patients: study design

**DOI:** 10.1093/eurpub/ckad133.250

**Published:** 2023-09-11

**Authors:** Martijn Bakker, Rhoda Schuling, Johan de Jong, Rienk Dekker

**Affiliations:** Hanze University of Applied Sciences, The Netherlands; University of Groningen (RUG); University Medical Centre Groningen (UMCG); ma.bakker@pl.hanze.nl; Hanze University of Applied Sciences, The Netherlands; Hanze University of Applied Sciences, The Netherlands; University of Groningen (RUG); University of Groningen (RUG); University Medical Centre Groningen (UMCG); ma.bakker@pl.hanze.nl

## Abstract

**Purpose:**

Physical inactivity is a major public health concern and a growing problem among stroke patients. Despite existing interventions, current approaches are not successful in promoting physical activity sustainably in stroke patients, particularly when transitioning from clinical care to the home environment. In co-creation with patients and healthcare professionals, this study develops an active lifestyle intervention, tailored to patients' personal, social, and environmental determinants, to promote an active lifestyle in their own living environment.

**Methods:**

A co-creative design-based approach will be used for the development of an active lifestyle intervention. The design process involved collaboration with stroke patients, physiatrists, physiotherapists, lifestyle coaches and health behavior professionals. A classic design thinking process will be used to structure the research activities in five phases: empathize, define, ideate, prototype, and test phases. To gather the needs and requirements for the intervention, interviews (n = 15) and focus group sessions (n = 15) will be held. An iterative process of prototyping will lead to a final version of the active lifestyle intervention.

**Results:**

At the conference, our team will be able to present the design of the study, focusing in particular on the aspect of engaging patients to co-create the design-based intervention so that patients’ personal, social and environmental determinants for sustained behavior change in the home environment can be adhered to.

**Conclusion:**

Designing an active lifestyle intervention together with patients and professionals, using the co-creative design-based approach, will help to gain deeper insights in the specific needs and requirements of stroke patients transitioning from clinical care to the home environment.

